# Subtotal resection and adjuvant radiotherapy for a fourth ventricle rosette-forming glioneuronal tumor in a child: a case report and review of management strategies

**DOI:** 10.1097/MS9.0000000000005019

**Published:** 2026-05-14

**Authors:** Oualid Mohammed Hmamouche, Marouane Hammoud, Badereddine Mohammadine, Faycal Lakhdar, Mohammed Benzagmout, Khalid Chakour, Mohammed El Faiz Chaoui

**Affiliations:** Department of Neurosurgery, Hassan II University Hospital of Fez, Morocco

**Keywords:** case report, child, fourth ventricle, glioneuronal, RGNT, rosette-forming

## Abstract

**Introduction and importance:**

Rosette-forming glioneuronal tumor (RGNT) is a rare World Health Organization grade I tumor of the central nervous system, most commonly found in the fourth ventricle. Preoperative diagnosis is challenging, and optimal management for incompletely resected tumors is not well-established.

**Case presentation:**

An 8-year-old boy presented with headaches and declining vision due to obstructive hydrocephalus caused by a large fourth ventricle tumor with extension into the cerebellopontine angle. After an endoscopic third ventriculostomy, the patient underwent maximal safe subtotal resection. Histopathological examination confirmed RGNT. Given the residual tumor, adjuvant radiotherapy was administered. Following treatment, the patient’s symptoms resolved, and follow-up imaging demonstrated stable residual disease.

**Clinical discussion:**

Although RGNTs are indolent tumors, their location in the fourth ventricle often precludes complete resection. Subtotal resection followed by adjuvant radiotherapy *may represent a valid option in selected cases* where complete resection is not feasible, with the aim of balancing tumor control and neurological preservation. This case adds to the limited pediatric experience and emphasizes the importance of a personalized, multidisciplinary approach with careful long-term surveillance.

**Conclusion:**

This case highlights the importance of *individualized, multidisciplinary decision-making* in the management of pediatric RGNTs of the fourth ventricle when complete resection is not feasible.

## Introduction

Rosette-forming glioneuronal tumor (RGNT) is a rare and distinct glioneuronal neoplasm, first described in 2002 and currently classified as a World Health Organization (WHO) grade I tumor^[^1,2^]^. It predominantly affects young adults and is typically located in the posterior fossa, with the fourth ventricle being the most common site^[^3^]^. Histologically, RGNT is characterized by a biphasic architecture, combining neurocytic rosettes and a glial component resembling pilocytic astrocytoma.

Preoperative diagnosis of RGNT remains challenging, as its radiological features often overlap with those of other posterior fossa tumors, including ependymoma and pilocytic astrocytoma^[^4^]^. Although gross total resection (GTR) is widely regarded as the optimal therapeutic goal, this objective may not always be achievable without unacceptable neurological risk, particularly in pediatric patients when the tumor is adherent to the floor of the fourth ventricle or extends toward the cerebellopontine angle. Consequently, the management of residual disease following subtotal resection (STR) remains controversial.

HIGHLIGHTS

- Rare case of pediatric rosette-forming glioneuronal tumor (RGNT).
- Subtotal resection was achieved after an endoscopic third ventriculostomy.
- Adjuvant radiotherapy provided durable control of the residual tumor.
- Multidisciplinary approach enabled full symptom resolution.
- Long-term surveillance remains essential in pediatric RGNT.

Surgical resection remains the cornerstone of treatment for RGNT and is generally associated with favorable outcomes when gross total resection can be achieved. However, as these tumors frequently arise in the fourth ventricle and may adhere to the brainstem or cerebellar structures, complete resection is not always feasible without significant neurological risk. In such situations, careful radiological surveillance is often recommended, although adjuvant therapies such as radiotherapy, chemotherapy, or stereotactic radiosurgery have occasionally been reported in selected cases^[^3,5–8^]^.

Here, we report a pediatric case of fourth ventricle RGNT, in which anatomical constraints precluded complete resection, leading to an individualized, multidisciplinary decision-making process. This case is presented alongside a focused narrative review of reported management strategies. A focused narrative review of the literature was performed using PubMed and Google Scholar, targeting reports of histologically confirmed RGNT with available surgical and outcome data. Given the rarity of pediatric RGNT and the heterogeneity of published cases, a systematic review or meta-analysis was not considered feasible.

This work has been reported in line with the SCARE 2025 criteria^[^9^]^.

## Case report

**Figure 1.**
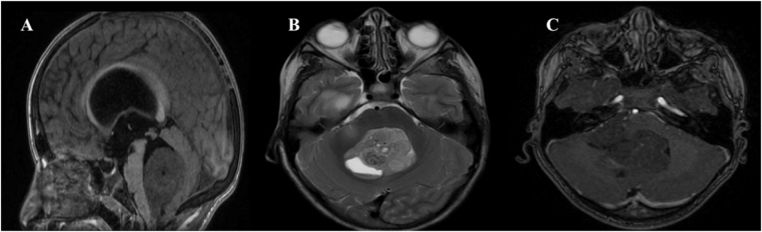
Preoperative MRI of a fourth ventricular tumor. (A) Sagittal T1-weighted image showing a midline posterior fossa mass filling and expanding the fourth ventricle. (B) Axial T2-weighted image demonstrating heterogeneous hyperintensity with a central cystic component. (C) Axial post-contrast T1-weighted image showing heterogeneous enhancement, with secondary compression of the cerebellar vermis and brainstem.

An 8-year-old boy presented with a 3-month history of progressive headaches and declining vision. Neurological examination revealed bilateral papilledema. Brain magnetic resonance imaging (MRI) demonstrated a large, heterogeneously enhancing solid-cystic mass measuring 20 × 33 × 45 mm, centered in the fourth ventricle. The lesion extended through the right foramen of Luschka into the cerebellopontine angle and was responsible for severe obstructive hydrocephalus (Fig. 1).

The patient initially underwent an emergency endoscopic third ventriculostomy (ETV) to relieve hydrocephalus, which resulted in the rapid resolution of acute symptoms. He subsequently underwent a suboccipital craniotomy via a transvermian approach for tumor resection. Approximately 95% of the tumor volume was resected. Complete resection was intentionally avoided due to the firm adherence of the residual tumor to the floor of the fourth ventricle, where further dissection carried a high risk of brainstem injury.

Intraoperative neuromonitoring was not available; however, transient cardiorespiratory rhythm disturbances observed during intraoperative monitoring reinforced the decision to prioritize neurological safety and terminate further resection.

**Figure 2.**
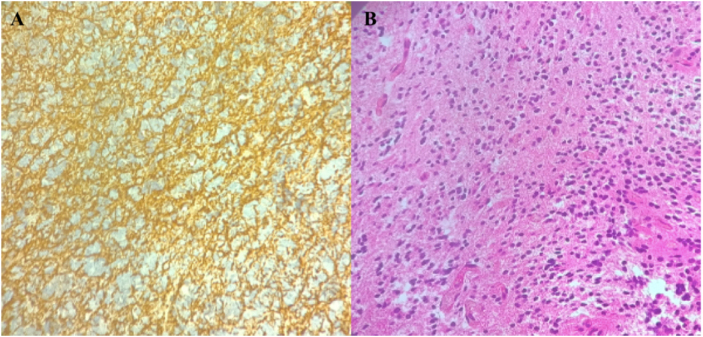
Histopathology of the glial component of RGNT. (A) Hematoxylin and eosin–stained section showing the glial component of the tumor with pilocytic astrocytoma–like features. (B) Immunohistochemistry demonstrating strong GFAP expression in the glial areas.

**Figure 3.**
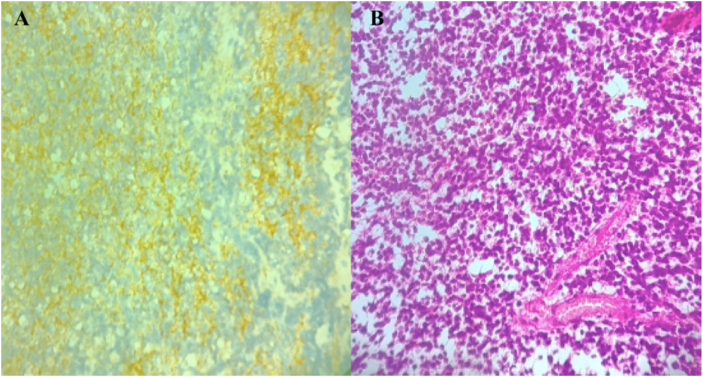
Histopathology of the neurocytic component of RGNT. (A) Immunohistochemistry showing strong synaptophysin expression in the neurocytic rosettes. (B) Hematoxylin and eosin–stained section demonstrating uniform neurocytic rosettes and perivascular pseudorosettes in a mucinous background.

Histopathological examination revealed a biphasic tumor composed of a glial component resembling pilocytic astrocytoma (Fig. 2) and a neurocytic component characterized by small, uniform cells forming neurocytic rosettes and perivascular pseudorosettes (Fig. 3), consistent with the diagnosis of RGNT. The Ki-67/MIB-1 labeling index was approximately 10%, indicating higher proliferative activity than typically reported for indolent RGNT. Molecular testing for BRAF or FGFR1 alterations was unavailable and therefore not performed.

**Figure 4.**
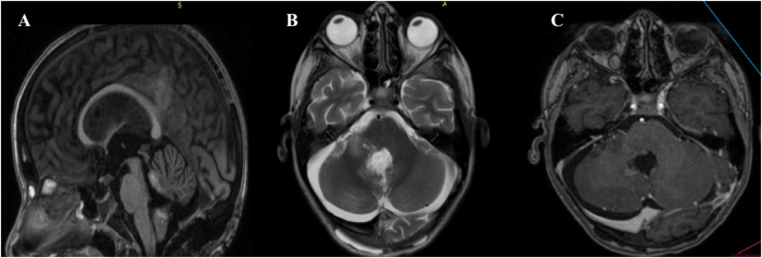
Postoperative MRI after suboccipital craniotomy for a fourth ventricular tumor. (A) Sagittal T1-weighted post-contrast image showing the resection cavity in the fourth ventricle. (B) Axial T2-weighted image demonstrating postoperative changes and a small residual lesion in the cerebellopontine angle. (C) Axial T1-weighted post-contrast image confirming residual enhancing tissue adjacent to critical neurovascular structures.

Postoperative MRI confirmed successful tumor debulking, with a small residual lesion located in the right cerebellopontine angle (Fig. 4). The patient recovered well from surgery without new neurological deficits.

Given the presence of residual tumor in a critical brainstem-adjacent location and the elevated proliferative index, the case was discussed at a multidisciplinary tumor board. Although active surveillance is a commonly adopted strategy following STR of RGNT, adjuvant radiotherapy was selected as a risk-balanced option in this specific context. Advanced radiotherapy techniques were not available in our healthcare setting, and conventional cranial radiotherapy represented the only accessible modality. At the 12-month follow-up, the patient demonstrated complete clinical recovery and short-term radiological stability of the residual tumor on serial MRI.

## Discussion

RGNT is a rare, low-grade glioneuronal neoplasm, but its management can be challenging, particularly when complete surgical resection is not feasible. The present case illustrates the diagnostic and therapeutic difficulties associated with this tumor and highlights the complexity of decision-making in the setting of residual disease.

The primary diagnostic challenge of RGNT lies in its nonspecific radiological appearance, which frequently overlaps with that of other posterior fossa tumors, including ependymoma and pilocytic astrocytoma. As a result, histopathological examination remains essential for establishing a definitive diagnosis^[^4^]^.

Although gross total resection is widely accepted as the primary therapeutic objective in RGNT, the optimal management strategy following subtotal resection remains less clearly defined. This uncertainty is particularly relevant for tumors arising in the fourth ventricle, where aggressive resection may carry a substantial risk of neurological morbidity. To contextualize our case, we have summarized selected reported cases of RGNT in Table 1.

**Table 1:** Summary of selected rosette-forming glioneuronal tumor (RGNT) case reports.

Author(s) & year	Age	Sex	Tumor location	Presenting symptoms	Extent of resection	Adjuvant therapy	Follow-up	Outcome
Komori et al, 2002^[^1^]^	24	F	Fourth ventricle	Ataxia, dizziness	Gross total (GTR)	None	20 months	No recurrence
Arai et al, 2010^[^10^]^	46	M	Fourth ventricle	Headache, nausea	Gross total (GTR)	None	12 months	No recurrence
Bera et al, 2017^[^11^]^	13	M	Cerebellar vermis	Headache, vomiting	Subtotal (STR)	None (Observation)	20 months	Stable residual
Morris et al, 2017^[^5^]^	6	M	Fourth ventricle	Not reported	Subtotal (STR)	Chemotherapy	>24 months	Progression, then GTR
Wilson et al, 2020^[^3^]^	12	M	Fourth ventricle	Headache, dizziness	Subtotal (STR)	Radiotherapy	60 months	Stable residual
Cacciotti et al, 2021^[^12^]^	9	F	Cerebellar vermis	Headache, vomiting	Gross total (GTR)	Proton Therapy	>36 months	Two recurrences, stable post-PBT
Huang et al, 2022^[^6^]^	26	F	Fourth ventricle	Headache	Gross total (GTR)	None	18 months	No recurrence
Liu et al, 2024 (Cases)^[^7^]^	35*	M/F	Various	Various	GTR/STR	None	4–23 months	No progression/recurrence
Shen et al, 2024^[^8^]^	35	F	Pineal region & brainstem	Syncope	Partial resection	Gamma Knife	3 months	Significant shrinkage
Hmamouche et al	8	M	Fourth ventricle & CPA	Low vision, Headaches	Subtotal (STR)	Radiotherapy	Not Reported	Stable residual

As illustrated in Table 1, management strategies after subtotal resection are heterogeneous. Although observation with serial imaging is frequently adopted and may be appropriate in many cases, adjuvant treatments such as radiotherapy, chemotherapy, or stereotactic radiosurgery have been reported in selected situations. These variations reflect the absence of standardized guidelines and the need to tailor treatment decisions based on anatomical constraints, clinical presentation, and biological tumor characteristics.

In the present case, anatomical considerations precluded gross total resection without exposing the patient to an unacceptable neurological risk. The multidisciplinary decision to combine near-total resection with adjuvant radiotherapy was, therefore, guided by a careful balance between tumor control and functional preservation, rather than an intention to propose a standard treatment approach.

Although RGNT is classified as a WHO grade I tumor^[^2^]^, its biological behavior is not uniformly indolent. Reports of recurrence or progression, even after gross total resection, have been described in the literature. Notably, the case reported by Cacciotti *et al*^[^12^]^, which recurred twice despite initial complete resection, underscores the potential for unpredictable behavior. This variability supports the rationale for individualized management strategies and highlights the importance of long-term radiological surveillance in all patients.

Recent studies have also highlighted the importance of molecular alterations in RGNT. Genetic abnormalities, such as *KIAA1549–BRAF fusion* and other molecular alterations, have been reported in some cases and may contribute to tumor development and biological behavior^[^4,5^]^. Molecular characterization may, therefore, provide additional insights into tumor pathogenesis and could potentially influence therapeutic strategies in the future. In the present case, molecular testing for BRAF or other alterations was not available at our institution and, therefore, could not be performed.

## Conclusion

In this specific case, maximal safe subtotal resection, followed by carefully selected adjuvant radiotherapy, resulted in short-term clinical and radiographic stability, underscoring the importance of individualized decision-making when complete resection is not feasible.

## Data Availability

Data sharing is not applicable to this article, as no new datasets were generated or analyzed during the current study.
